# Interferometry and Simulation of the Thin Liquid Film between a Free-Rising Bubble and a Glass Substrate

**DOI:** 10.1021/acs.langmuir.1c03374

**Published:** 2022-02-07

**Authors:** Ivan U. Vakarelski, Kenneth R. Langley, Fan Yang, Sigurdur T. Thoroddsen

**Affiliations:** † Division of Physical Sciences and Engineering, King Abdullah University of Science and Technology (KAUST), Thuwal 23955-6900, Saudi Arabia; ‡ Department of Mechanical, Aerospace and Biomedical Engineering, University of Tennessee Space Institute, Tullahoma, Tennessee 37388, United States

## Abstract

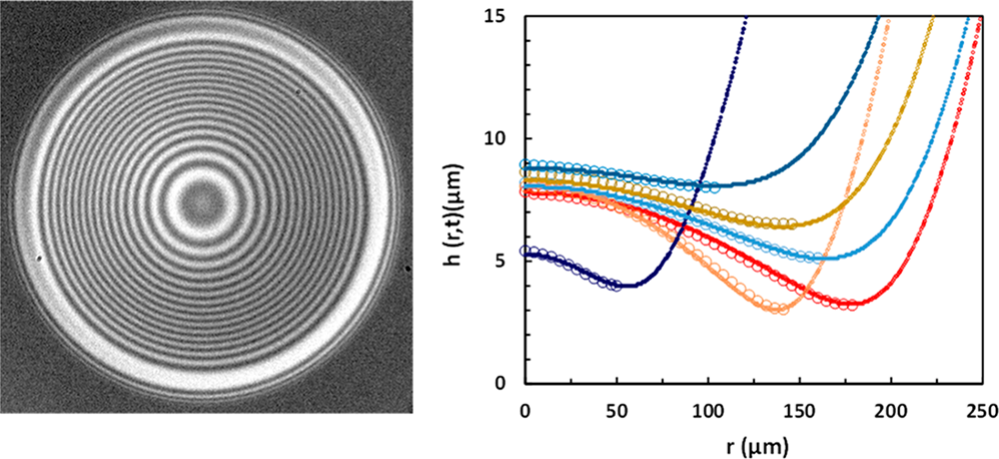

Because of their practical importance and complex underlying physics, the thin liquid films formed between colliding bubbles or droplets have long been the subject of experimental investigations and theoretical modeling. Here, we examine the possibility of accurately predicting the dynamics of the thin liquid film drainage using numerical simulations when compared to an experimental investigation of millimetric bubbles free-rising in pure water and colliding with a flat glass interface. A high-speed camera is used to track the bubble bounce trajectory, and a second high-speed camera together with a pulsed laser is used for interferometric determination of the shape and evolution of the thin liquid film profile during the bounce. The numerical simulations are conducted with the open source Gerris flow solver. The simulation reliability was first confirmed by comparison with the experimental bubble bounce trajectory and bubble shape evolution during the bounce. We further demonstrate that the simulation predicted time evolution for the shape of the thin liquid film profiles is in excellent agreement with the high-speed interferometry measured profiles for the entire experimentally accessible film size range. Finally, we discuss the implications of using numerical simulation together with theoretical modeling for resolving the complex processes of high velocity bubble and droplet collisions.

## Introduction

Interactions involving bubbles and droplets are ubiquitous in many naturally occurring and biological processes as well as in industrial processes and products.^1^ When bubbles or droplets collide or approach an interface, a thin liquid film is formed between the two bubbles or the bubble and the interface due to the deformability of the liquid interface. The draining dynamics of the thin liquid film determines the behavior of many practically important gas or droplet emulsion systems. Because of their practical importance and the diverse underlying physics, the dynamics and the stability of the thin liquid film have been extensively studied in the past both experimentally and using theoretical models.^2−12^ However, so far there have been only limited attempts to investigate the dynamics of the thin liquid film drainage using numerical simulations.^13^ In this paper, we aim to evaluate the reliability of the numerical simulation in predicting the thin liquid film dynamic behavior by comparing the simulated thin liquid film profiles with interferometrically measured thin liquid film profiles.

In recent studies, we have examined the interface mobility effect on the dynamics of free-rising bubble collisions with various liquid interfaces.^14−17^ A clean liquid–air interface is tangentially mobile, whereas contamination or the presence of surfactant can immobilize the interface due to Marangoni effects.^18,19^ Bubbles with mobile interfaces are expected to coalesce much faster than bubbles with immobile interfaces because of the lower hydrodynamic resistance to the drainage of the thin liquid between the colliding bubbles.^11,14^ Together with the expected fast coalescence, our experiments demonstrated that mobile interface bubbles could bounce back much stronger from a mobile liquid interface compared to an immobile liquid interface.^15,16^ The general explanation of the stronger bounce is in the lower viscous dissipation during collisions involving mobile liquid interfaces compared to immobile liquid interfaces.

The surface mobility effects in our studies were confirmed by numerical simulation of the free-rise and subsequent bounce of bubbles from the interface.^15,16^ Prior to our investigations, the bubble free-rise and bounce from liquid interfaces were also studied with an analytical force balance model that accounts for buoyancy, hydrodynamic drag, added mass, and film forces.^20−22^ However, because of the complexity of the problem, the model uses several simplifications, for example, for the variation of the added mass force and bubble shape near the interface, which in practice might act as adjustable parameters.^23^ At the same time, our numerical simulations showed good agreement with the experiments for the bubble bounce trajectories and bubble shape variation without the use of any fitting parameters.^15,16^ In the present work, we aim to extend these investigations by examining if the numerical simulations can correctly reproduce the profiles of the thin liquid film formed during the bouncing of a free-rising bubble from the interface. The numerical simulation profiles will be compared with film profiles measured using high-speed interferometry.^24,25^

Because of the technical difficulties related to the interferometric measurement during the bubble bounce from a free liquid interface, we conduct our investigation for bubble bounce from a transparent solid interface. The system we chose comprises millimeter size bubbles free-rising in water and bouncing from a flat glass substrate. Similar interferometric measurements used to evaluate the thin liquid film during the impact of a water bubble on glass have been conducted before by Hendrix et al.^24^ However, due to the presence of trace contaminates, the measurements in that work were limited to bubbles with a rise velocity consistent with that of a bubble with an immobile interface. In the present investigation, we manage to conduct experiments with air bubbles in water with a free-rise velocity as well as the bounce from the glass interface trajectory, which were in excellent agreement with the prediction for bubbles with fully mobile interfaces. By conducting experiments using bubbles with clean interfaces, we avoid the ambiguity of the data interpretation due to the presence of trace contamination in the system.^26^

## Experimental and Numerical Methods

### Experimental Setup

**Figure 1:**
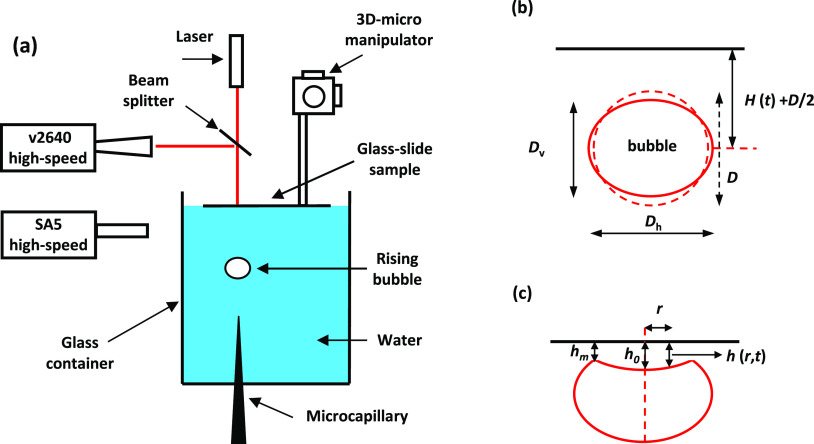
(a) Schematic of the experimental setup. (b) Schematic of an oblate ellipsoidal bubble of horizontal diameter *D*_h_ and vertical diameter *D*_v_ approaching the glass sample. The undeformed bubble of diameter *D* = (*D*_h_^2^*D*_v_)^1/3^ is indicated by the red dashed line. The bubble center-of-mass position, relative to the glass sample reference position, is *H*(*t*) as indicated, with *H*(*t*) = 0 corresponding to the undeformed bubble touching the glass sample. (c) Schematic of a bubble bouncing from the glass substrate, with *h*(*r*,*t*) tracking the thin liquid film profile shape.

The experimental setup used to monitor the bubble free-rise and bounce from the glass together with the laser interferometry of the bubble bounce from the glass substrate is schematized in Figure 1a. The glass container was an optical glass cell (Hellma Analytics), with a cross section of 15.0 × 5.5 cm and a height of 10.0 cm. A small hole was drilled through the bottom of the cell, into which a glass microcapillary of 100 μm inner diameter was inserted. The capillary is connected by a plastic tube to a pressure regulator used to generate controlled air-flow pulses. Using combinations of different air pressure and pulse duration, we were able to release bubbles with diameters in the range of 0.6 to 1.6 mm. A glass slide was mounted on the holder of a 3D micromanipulator, which was used to slowly lower the slide into contact with the water interface.

The side-view of the bubble free-rise and bounce from the glass interface was recorded with a high-speed camera, Photron-SA5. The typical filming rate used is 5,000 frames per second (fps). The camera was equipped with a 5× microscope objective giving an image resolution of 4 μm/pixel. The time trajectories of the bubble center-of-mass position were extracted by processing the videos using an in-house developed MATLAB image processing code.

A second high-speed camera, Phantom v2640, was used to record the interferometric patterns during the bubble bounces from the glass substrate. The typical recording rate was 6,600 to 45,000 fps. This camera is equipped with a long-distance microscope (Leica Z16 APO) with adjustable magnification yielding a spatial resolution of up to 0.9 μm/pixel. A pulsed laser diode, SILUX-640 (Specialised Imaging), with a wavelength of 640 nm and a pulse duration between 10 and 100 ns was positioned above the solid interface and used as the interference light source. A 50:50 beam splitter angled at 45° to the surface allowed the transmission of the laser light to the interface and the subsequently reflected interference to be imaged on the camera.

### Bubble Bounce from Glass Experiments

The water used was purified in a Millipore apparatus, with an internal specific electrical resistance of no less than 18.4 MΩ/cm. Before the experiments, the glass vessel was plasma cleaned and washed with copious amounts of deionized water. The glass samples used were Fisher cover glass slides. The glass slides were very smooth with an atomic force microscopy determined RMS of about 1.2 nm.^27^ After washing with ethanol and water, the glass is hydrophilic with an advancing water contact angle of less than 30°.

Bubbles with an undeformed diameter between 0.8 and 1.5 mm were studied. For this size range, the free-rising bubbles assume an oblate ellipsoidal shape, as shown in Figure 1b. It is convenient to characterize the bubble using the equivalent diameter, *D* ≡ (*D*_h_^2^*D*_v_)^1/3^, where *D*_h_ and *D*_v_ are the horizontal and vertical ellipsoidal diameters. In all experiments, the bubbles were released from at least 2.5 cm below the water–glass surface to ensure that the bubbles reached terminal velocity before reaching the interface. The position of the bubble center-of-mass through time, *H*(*t*), is measured relative to the glass sample surface, with *H* = 0 corresponding to the undeformed bubble touching the sample (Figure 1b).

Bubble bounce trajectories and interferometric measurements of the bubble bounce from the substrate were done in separate runs. In all cases, we had very good reproducibility for both the bounce trajectory and interferometric patterns when identical size bubbles were used. In the runs when the bubble trajectory was measured, the side-view camera was focused close to the glass interface to capture the bubble bounces from the glass. In the case when we were recording the interferometric patterns of the bubble bounces from the glass substrate, the side-view camera was focused close to the microcapillary bubble release end to record the precise bubble size. Because the bubbles tend to stick to the glass substrate after impact, after each run, we use the micromanipulator to horizontally shift the glass slide to ensure that the next bubble will impact on a clean spot away from bubbles that attached in prior runs.

### Interferometric Measurement

The intensity of the reflected interference pattern, *I*(*r*), when the incident light is perpendicular to the interfaces, is dependent on the gap thickness between the air–water interface and the water–glass interface, *h*(*r*); the index of refraction of the intervening film, *n* = 1.33 for water; and the wavelength of the incident light, λ = 640 nm:
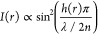
1

Intensity profiles were extracted from the acquired interference images along a selected row of pixels and averaged with the 3 rows above and below to reduce noise. Extrema were identified in the intensity profile and then converted to physical thicknesses by solving for *h*(*r*) in (1). Since only monochromatic light is used, this method gives the relative thickness between the perimeter of the deformation (minimum film thickness, *h*_m_) and the center of the deformation (maximum film thickness, *h*_0_) but not the absolute thickness of the film (Figure 1c). In our configuration, the resolution between extrema in adjacent bright and dark fringes is 120 nm.

### Gerris Numerical Simulation (GNS)

As in our recent work on bubbles bouncing from interfaces in a perfluorocarbon liquid PP1,^15^ ethanol, or water,^16^ we conducted numerical simulations using the freely available open-source code Gerris flow solver.^28−31^ This code uses the volume-of-fluid (VOF) method to solve the incompressible Navier–Stokes equations. Because the code is easy to adapt for an axisymmetric geometry and uses a local adaptive mesh approach, the code is very efficient for the simulation of bubble and droplet collisions with an interface.

Supporting Figure S1 shows the dimensions of the simulation domain used. We use a large enough simulation domain to minimize the effect of the finite size of the domain. The simulation uses the nominal physical parameters of the system: water density is 997.8 kg/m^3^, and water viscosity is 1.00 mPa s. Air density is 1.21 kg/m^3^, and viscosity is 1.81 × 10^–2^ mP s. The water–air surface tension was set to 72.4 mN/m.

The generic Gerris code allows application of both the no-slip (immobile) and the free-slip (fully mobile) boundary conditions at the top wall. All simulations start with an adaptive mesh with level 11 maximum refinement, i.e., the axisymmetric planar domain is split into squares, where a localized refinement step splits a square into half in both directions. The size of the smallest cell is therefore 2^11^ times smaller than the original domain. The refinement is performed based on the distance from the interface, as well as the amplitude of the velocity and vorticity gradients. During the bubble approach and bounce from the interface, the refinement level is gradually increased to resolve the thin liquid film between the bubble and the interface. In the case of a no-slip top-wall boundary condition, the additive mesh level needs to be increased to level 14, to resolve the first two bounces of the bubble from the interface. For some cases using the free-slip top wall boundary condition, we need to increase the adaptive mesh level up to 17, which corresponds to the smallest cell being reduced by 2^17^ to ∼75 nm. The thinnest liquid films are always resolved by at least 2–3 cell widths. Each simulation has been run using 20 cores in parallel within the KAUST IBEX cluster computer nodes (Intel Xeon Gold 6148 Processors), and the computational time was from 2 to 22 days.

## Results and Discussion

### Bubble Bounce Trajectory

First, we examine the free-rise velocity and the center-of-mass versus time dependence for the free-rising bubbles bouncing from the glass interface. The terminal velocity of the free-rising bubbles is used to confirm that in our experiments the bubbles behave as fully mobile interface bubbles. The comparison of the bounce from the interface trajectory is used to evaluate the general consistency between the experiment and the Gerris numerical simulation (GNS).

For the water bubble sizes investigated here, the fully mobile bubble terminal rise velocity was estimated using the Moore theory for high Reynolds number deformable bubbles.^20−22,32^Supporting Figure S2 compares the bubble free-rise velocities measured in our experiments with the prediction of the Moore theory. The results are in excellent agreement with the theoretical prediction confirming that, as in our prior investigation for bubbles bouncing from free water–air interfaces, the bubble interface is mobile over the entire range of bubble sizes investigated.^16,33^

**Figure 2:**
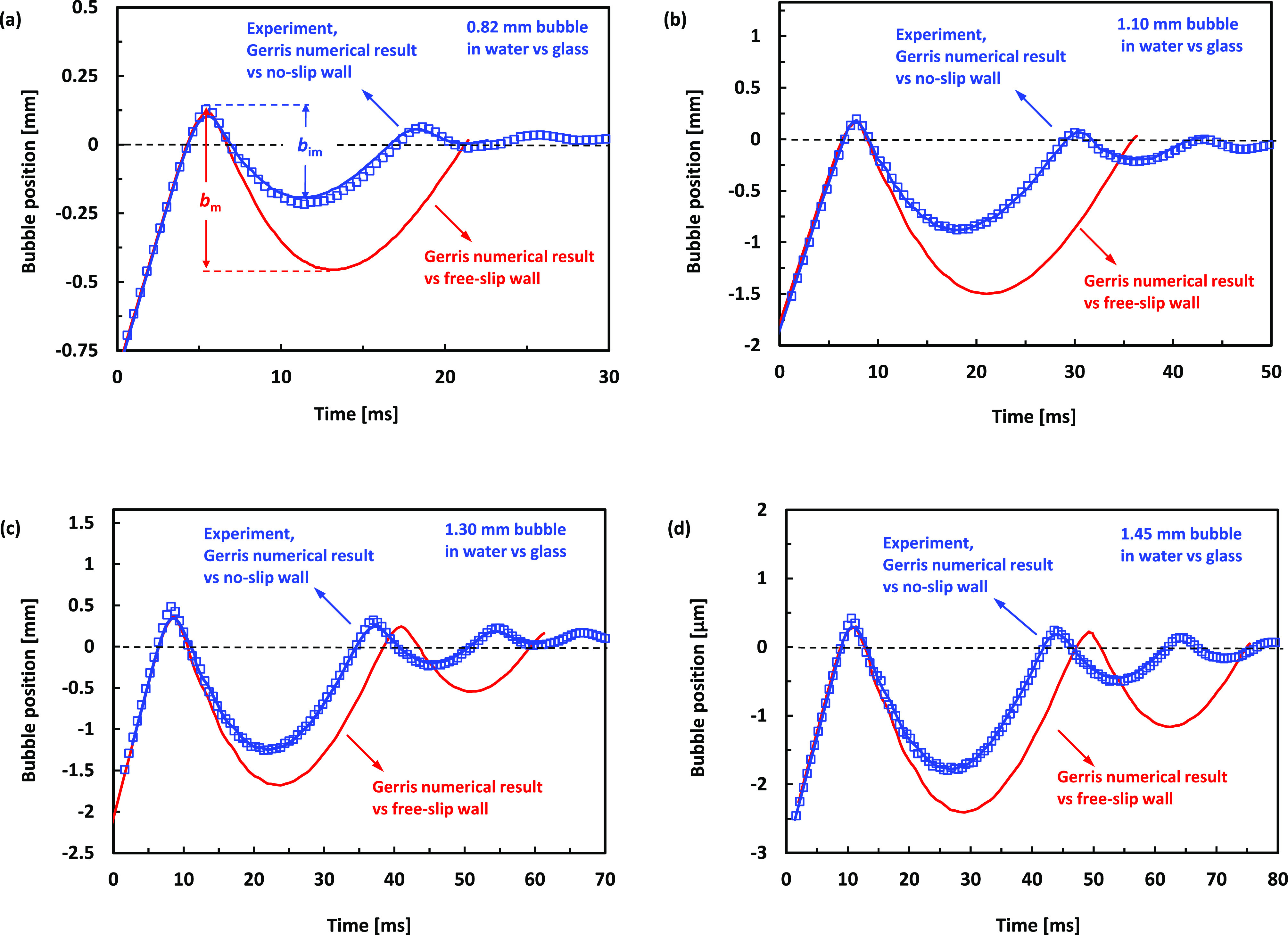
Experimental and GNS bubble center-of-mass positions versus time data for free-rising bubbles in pure water bouncing from a flat glass sample for (a) a *D* = 0.82 mm bubble, (b) a *D* = 1.10 mm bubble (Video 1 example), (c) a *D* = 1.30 mm bubble, and (d) a *D* = 1.45 mm bubble (Video 2 example). Empty blue squares are the experimental data, the solid blue line is the Gerris numerical result versus a no-slip wall, and the solid red line is the Gerris numerical result versus a free-slip wall.

**Figure 3:**
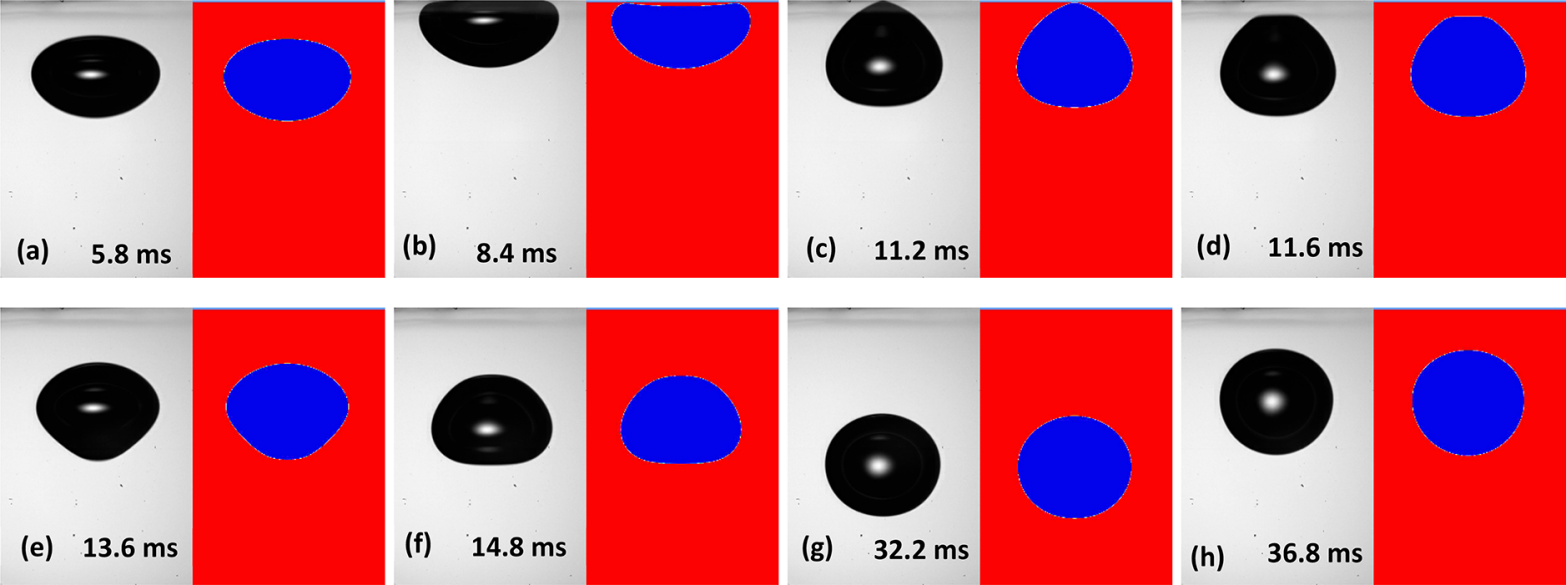
(a–h) Snapshots from Video 2 comparing the experimental video and GNS for a 1.45 mm bubble bounce from a glass substrate. Time in ms is given on each snapshot. Excellent agreement is demonstrated between experiment and simulation for the complex bubble shape evolution during the bounce from the interface.

Next, we compare the experimental bubble center-of-mass trajectories for the free-rising bubble bouncing from the glass substrate with the GNS predicted trajectories. The plots in Figure 2 compare the experimental and simulated trajectories for bubbles with undeformed diameters of 0.82 mm, 1.10 mm, 1.30 mm, and 1.45 mm. Supporting Video 1 is a combined video paralleling experiment and simulation for the case of a 1.10 mm bubble, and Video 2 is for the case of a 1.45 mm bubble. As seen in the videos and quantified in the Figure 2 plots for the entire range of bubble sizes investigated, we observe an excellent agreement between the experimental and simulated bubbles trajectories. The good agreement between simulation and experiment extends to the complex way in which the bubble shape is evolving during the bubble bounce from the interface. This is illustrated in Figure 3 via a snapshot series from Video 2 for the case of the 1.45 mm bubble bouncing from the flat solid interface.

In addition to the bubble bounce trajectories from a solid wall with a no-slip boundary condition, the plots in Figure 2 also show the GNS simulation for a bubble bouncing from a free-slip wall. These simulations confirm the stronger bounce from mobile interfaces compared to immobile interfaces as found in our recent studies.^15,16^ We notice that the simulated effect has a similar magnitude as the experimental effect for the bubble bounce from the mobile and immobile water–air interface. Quantified as the ratio between the first bounce distance in the mobile versus the immobile case, *b*_m_/*b*_im_, the effect varies from *b*_m_/*b*_im_ = 1.8 to 1.2 with increasing bubble size from 0.80 mm to 1.5 mm for the water–air interface^16^ and from 1.9 to 1.3 for the same bubble size range in the case of the water-wall interface.

### Thin Liquid Film Profile Evolution

**Figure 4:**
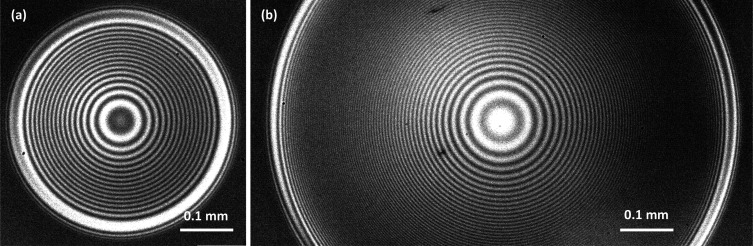
High-speed camera video snapshots of the interferometric patterns in the case of (a) a *D* = 0.80 mm free-rising bubble and (b) a *D* = 1.15 mm free-rising bubble, during the bubbles’ first bounce from the glass substrate. The pattern shown is at about the maximum film diameter extension. The image in (a) corresponds to the 0.75 ms data shown in Figure 5a, and in (b), the image corresponds to the 0.55 ms data shown in Figure 5b.

After confirming the agreement between simulation and experiment for the bubble trajectories and the bubble shape variation during the bounce from the interface, we next compare the simulation to interferometrically determined shapes of the thin liquid film during the bubble bounce from the glass. Because of the high free-rise velocity of the mobile interface bubbles and the relatively large bubble sizes used here, the interferometric measurement for the first bounce of the bubbles from the interface is challenging. The incremental difficulties of capturing the interferometric fringes with the increase in the bubble size are demonstrated in Figure 4. For the case of the first bounce of a *D* = 0.80 mm undeformed diameter bubble shown in Figure 4a, the pattern is clearly observed, whereas for the *D* = 1.15 mm bubble case shown in Figure 4b, we are approaching the limit at which the fringes can be resolved. We were able to resolve the first bounce of the free-rising bubble of undeformed diameters up to 1.20 mm, which were forming dimples of up to 1 mm in diameter and a depth of up to 15 μm. For comparison, most of the prior similar studies were limited to dimples with depths of less than one micron.^24^ At the same time because of the high impact velocity and the related high rates of the thin liquid film drainage, we were not able to resolve the absolute thickness of the thin liquid films. In essence, our comparison will be limited to the time evolution of the film profile shapes.

**Figure 5:**
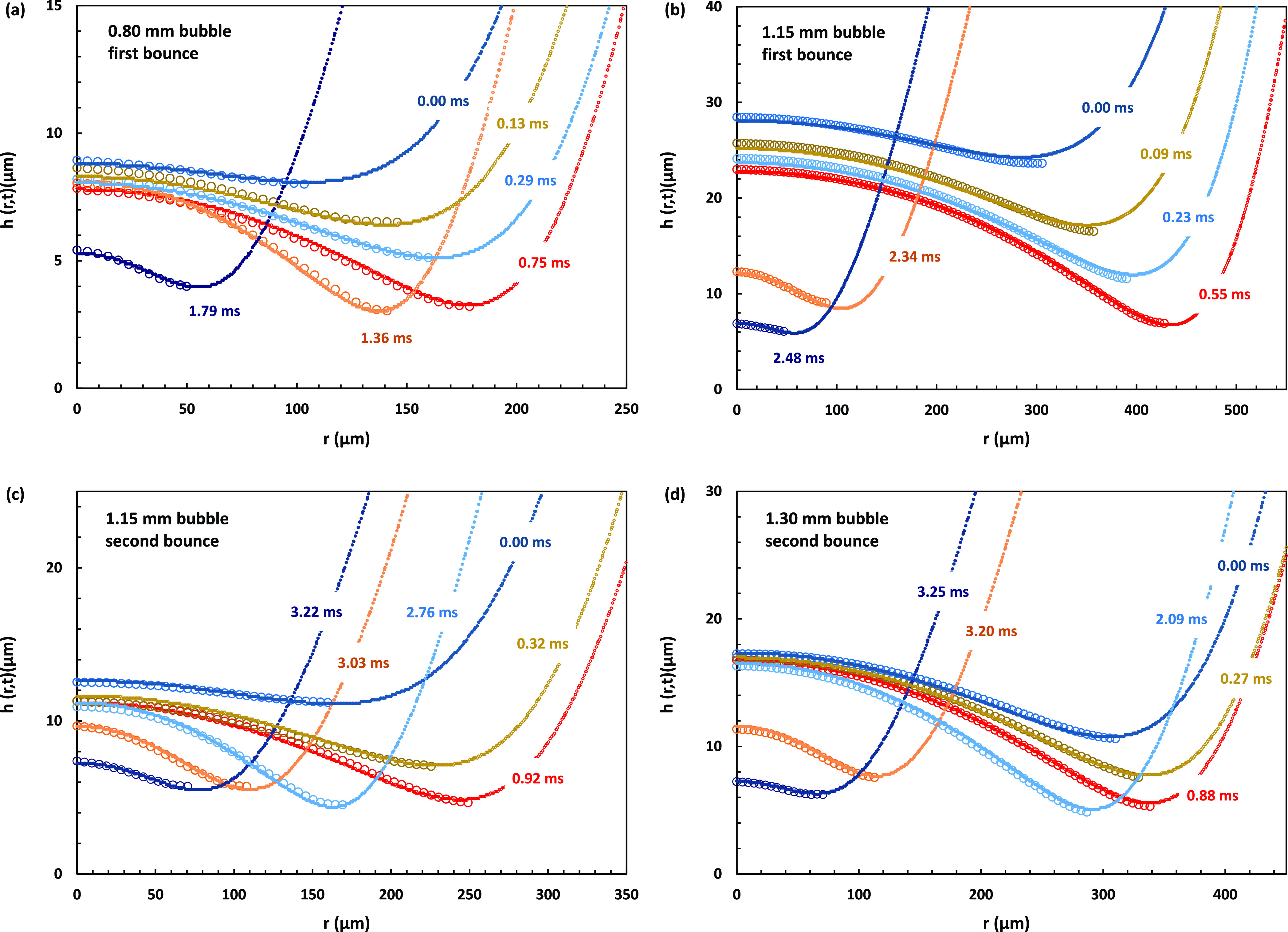
Comparison between film profiles obtained by interferometry (empty circles) and GNS (lines), film profiles for the cases of (a) a *D* = 0.80 mm bubble first bounce, (b) a *D* = 1.15 mm bubble first bounce, (c) the same *D* = 1.15 mm bubble second bounce, and (d) a *D* = 1.30 mm bubble second bounce from the glass surface. Only the right sides of the symmetric profiles are shown. Time in ms is indicated for each profile, with the zero-time set at the first profile to which the interferometry data are fitted. The red colored profiles corresponding to 0.75 ms in (a), 0.55 ms in (b), 0.92 ms in (c), and 0.88 ms in (d) are the film profiles with the maximum radial extent during the bubble approach to the interface, following which the bubbles start to retreat from the interface.

Comparing the time evolution of the film profiles shape, we obtained an excellent agreement between experiment and simulation, for both the first and the second bounce of the bubbles from the interface. In the Figure 5 plots, we compare simulation (lines) and interferometric profile data (empty circles) for four cases spanning the range of bubble sizes for which we resolved the first or the second bounce from the interface. Figure 5a shows the first bounce of a *D* = 0.80 mm bubble, Figure 5b shows the first bounce of a *D* = 1.15 mm bubble, Figure 5c shows the second bounce of the same *D* = 1.15 mm bubble, and Figure 5d shows the second bounce of a larger *D* = 1.30 mm bubble. As seen in the figure, in all these cases, the simulation was in very good agreement with the interferometrically measured profiles accurately tracking the shape of the dimple formed during the bubble approach and retraction from the interface.

Although our investigation shows excellent agreement between experiment and simulation for the time evolution of the thin liquid film profile shape, one limitation is that we do not measure the absolute thickness of the film. The film thickness could be resolved using multicolored interferometry, as done in recent studies for bubbles slowly approaching an interface,^34^ or droplets impacting onto a solid surface in air, where ultrahigh-speed interferometry was also used.^35^ However, due to the significant technical challenges present in the case of our fast-rising bubble collision with the interface, the extension to multicolored interferometry is saved for future investigation.

### Comparison between Analytical Modeling and Simulation of Bubble Collision

One important consideration is that our simulation does not account for the surface forces acting between the bubble and the solid interfaces, such as the van der Waals or the electric double layer forces. Such forces affect the film drainage dynamics for film thicknesses that are typically below 100 nm and determine the outcome of the collision, i.e., film breakage and coalescence or the formation of a stable liquid film.^36,37^ However, for our experimental range during the first and second bounce of the bubble, the estimated minimum film thickness is always well above 1 μm, and the interaction can safely be assumed to be purely hydrodynamic.

As mentioned in the Introduction, in prior studies, the collision of the free-rising bubble with a solid or liquid interface was analytically modeled using a force balance to determine the bubble center-of-mass trajectory.^20−22^ The force balance includes the gravitational force, the hydrodynamic drag force, the added mass force accounting for the inertia of the surrounding liquid, and the film force accounting for the lubrication pressure buildup in the thin liquid film during the bounce from the interface. The film force is incorporated using the Stokes-Reynolds–Young–Laplace model (SRYL model) developed by the Melbourne University group. Since the film thickness is much smaller than the film radius, the model uses the Stokes-Reynolds lubrication theory of thin film drainage in combination with the Young–Laplace equation of droplet or bubble deformations. In addition to the hydrodynamic forces and deformation, the model can include surface forces, which as discussed above are important at the later stage of the film drainage. A detailed description of the model and its applications can be found in the 2010 Chan et al.^6^ review paper, and more recent developments can be found in the 2021 Liu et al.^11^ review paper.

In numerous studies, the SRYL model was shown to accurately predict the interaction between deformable drops or bubbles, including the spatiotemporal thickness profiles of the thin liquid between a droplet or bubble and a solid interface.^37−39^ However, in most of these experiments, the bubbles or droplets are close to each other or close to the interface and are brought in contact in a controlled way, for example using a constant approach and retraction velocity. The bounce of free-rising bubbles from a free liquid or solid interface presents a significantly more challenging problem. In such cases, the initial conditions for the application of the SRYL model depend on the prehistory of the bubble approach including the surrounding liquid flow and bubble shape variation during the bounce. In our latest study of water bubbles bouncing from free mobile and immobile water interfaces, we demonstrate that the force balance model prediction could deviate significantly from the experimental bubble bounce trajectory.^16^ One could expect that the modeling difficulty in predicting the bubble bounce trajectory might also extend to the prediction of the thin liquid film drainage dynamics studied herein.

**Figure 6:**
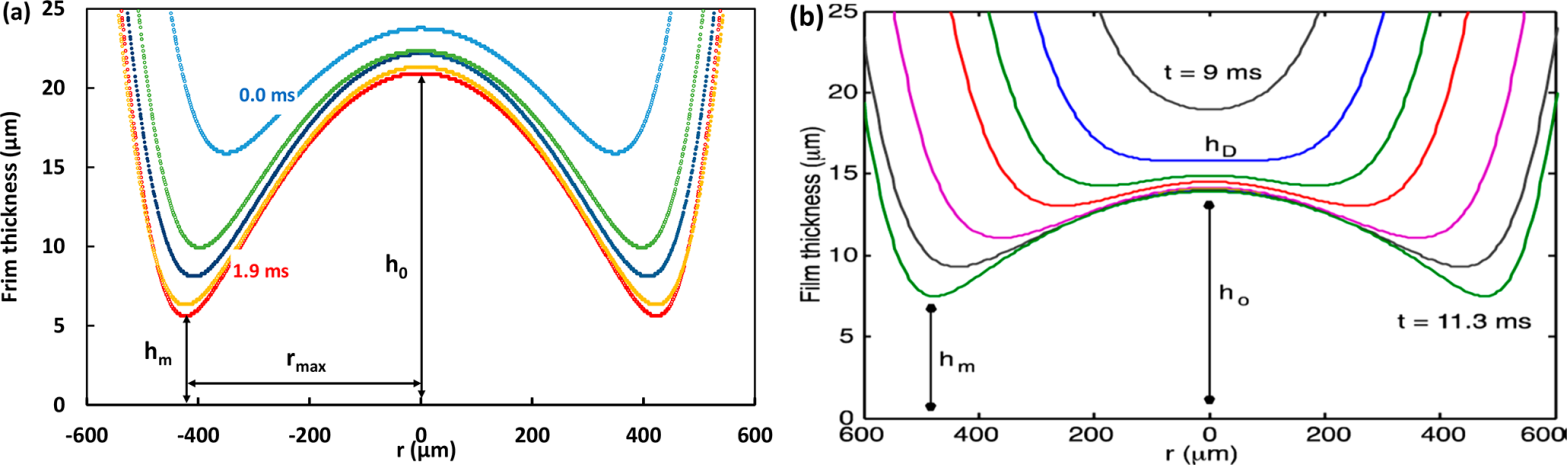
Comparison of (a) simulation and (b) modeling results for the thin liquid film profile shapes during the first approach to the wall of a *D* = 1.48 mm free-rising bubble in water. (b) is reproduced with permission from ref (20). Copyright 2015 American Chemical Society.

In Figure 6, we compare the spatiotemporal film thickness profiles for the case of a 1.48 mm free-rising water bubble bouncing from a flat solid interface obtained using our simulation and modeling profiles obtained using the force balance model as taken from Manica et al.^20^ Film profiles at an identical stage of dimple formation during the bubble’s first approach to the interface are compared. Both approaches predict similar values for the maximum film diameter, *r*_max_, and separation at the rim of the dimple, *h*_m_. However, the simulation predicts the formation of a significantly deeper dimple, (*h*_0_ – *h*_m_) ≈ 16 μm, compared to the model, (*h*_0_ – *h*_m_) ≈ 7 μm. The film formed during the first bounce for a *D* = 1.48 mm bubble exceeds the present work’s interferometric measurement range. Nevertheless, the excellent agreement between experiment and simulation for the shape of the dimple in all cases shown in Figure 5 indicates that the simulation prediction for the dimple shape in Figure 6 should be closer to the actual film shape than the modeling prediction.

The dimple formation during the bounce of the bubble from an interface, as observed in our measurements and simulations and shown in Figures 5 and 6, is a well-known phenomenon. However, our simulation implicates that for collision of larger bubbles, a more complex shape of the thin liquid film profile is possible. This is demonstrated in Supporting Video 3, which shows a simulation of the head-on collision of two bubbles. This simulation is done using the same approach as in our prior work to simulate the collision of mobile and immobile interface droplets.^15^ Due to the symmetry of the problem, the simulation of the head-on collision between two identical bubbles is identical to the simulation of the collision of a single bubble with a free-slip wall.

**Figure 7:**
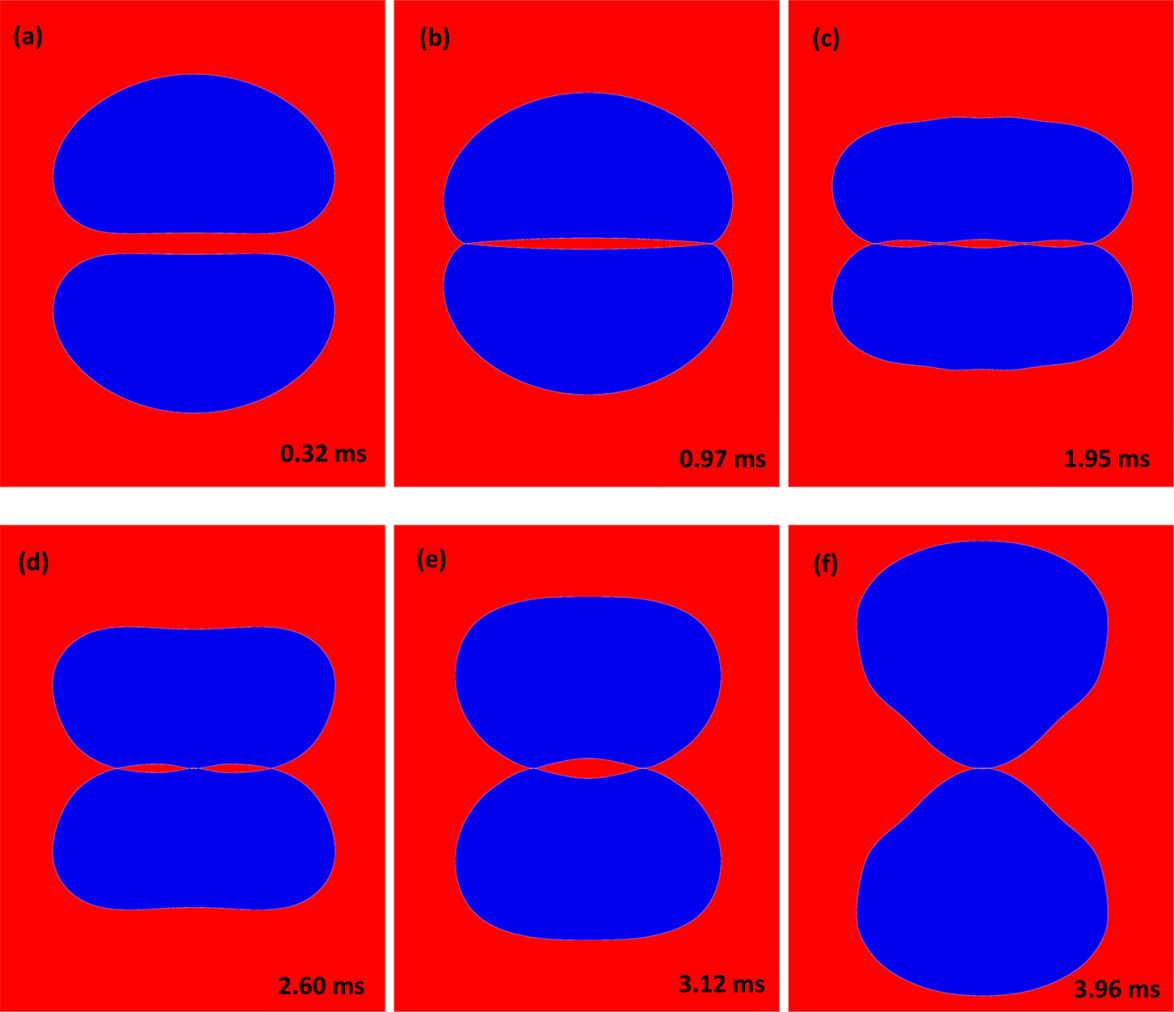
Snapshots from Video 3 showing a simulation of the head-on collision of *D* = 1.45 mm bubbles in pure water. The bubbles are initially accelerated by gravity to reach terminal velocity. Gravity is then removed at 0.1*D* bubble separation. Video time is shown in ms in each snapshot. Corresponding film profiles are featured in Figure 8.

**Figure 8:**
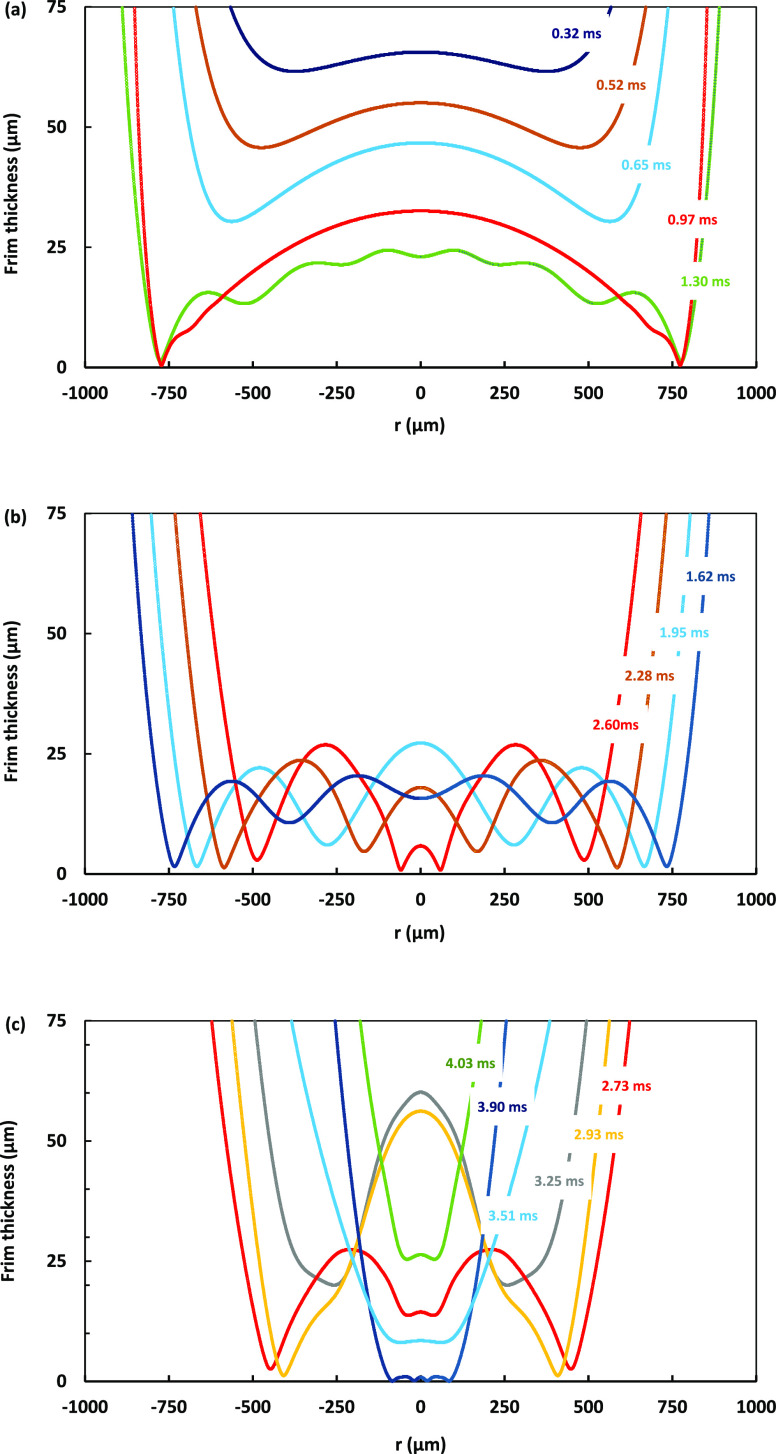
Simulated thin liquid film profiles for the head-on collision of two *D* = 1.45 mm bubbles in water shown in Video 3 and Figure 7 snapshots. Only the upper halves of the symmetric film profile are shown. Simulation time in ms is marked on each profile. (a) Dimple formation during the approach. (b) Complex shaped film with “double dimple” formation. (c) New dimple formation during the bounce back and separation.

In the Video 3 simulation, the *D* = 1.45 mm bubbles are accelerated using gravitational acceleration identically as in the Figure 2d free-rising bubble collision with a free-slip wall simulation; however, when the separation between the bubbles is equal to 0.1*D*, the gravity is switched off to simulate a head-on collision in the absence of any external forces. Snapshots from Video 3 featuring the major stages of the bubble collision are shown in Figure 7, and the corresponding film profile evolution is shown in Figure 8. After the initial formation of a large dimple (Figure 7b, Figure 8a), the film is progressing to a stage of double dimple formation (Figure 7c and 7d, Figure 8b), followed by a new dimple formation during the separation (Figure 7e, Figure 8c). Keep in mind that for the inverted configuration, of a drop impacting a glass surface in air, it is known that capillary waves can travel along the air–liquid interface, from the edge toward the center, where they can wet and pinch off a droplet.^40,41^ Here, the dominant inertia resides in the liquid film and prevents contact on the centerline. In any case, such complex behavior of the film drainage predicted by the simulation deviated significantly from the standard dimple formation during bubble and droplet collisions and needs to be confirmed experimentally in future investigations.

## Conclusions

By comparing experiments and simulations for free-rising bubbles bouncing from a glass surface, we demonstrate that numerical simulation can be used to predict not only the bounce trajectory together with the shape variation of the bubble during the collision but also the time variation of the shape of the intervening thin liquid film. For simpler situations such as the collision of two spherical bubbles or droplets in close proximity to each other, analytical models like the SRYL model provide a fast and accurate way to resolve the draining dynamics of the thin liquid film. Such models also have the advantage of easy integration of the surface forces that are important in the later stage of the film drainage. In contrast, for the case of large bubbles or droplets colliding at high speed, the deformation and the surrounding flow conditions can be too complex to be correctly resolved in detail analytically. In such cases, the high computational cost of numerical simulations seems justifiable, providing a more accurate prediction of the collision outcome including the thin liquid film drainage. Future efforts should be directed toward the development of more efficient computational approaches and for the integration of the computational and analytical methods. Including the effects of surface forces on the hydrodynamic forces in the simulation could be an important first step in that direction.

## Supporting Information Available

Figure S1, computational domain; Figure S2, free-rise velocity of bubbles in water (PDF)
Video 1, compares experiment (left) for *D* = 1.10 mm bubble bounce from glass interface with GNS result (right) for bubble bounce from no-slip wall (Time interval per frame is 0.2 ms (5000 fps), and the video is played at 30 fps.) (MP4)
Video 2, compares experiment (left) for *D* = 1.45 mm bubble bounce from glass interface with GNS result (right) for bubble bounce from no-slip wall (Time interval per frame is 0.2 ms (5000 fps), and the video is played at 30 fps.) (MP4)
Video 3, simulation for collision of two bubbles of *D* = 1.45 mm undeformed diameter (Initial bubbles acceleration is done using gravity field, which is switched off at 0.1*D* bubbles separation. Simulation time interval per frame is 0.65 ms, and the video is played at 30 fps.) (MP4)
