# Supplementary material for: Interferometry and Simulation of the Thin Liquid Film between a Free-Rising Bubble and a Glass Substrate
Source: Langmuir. 2022 Feb 7;38(7):2363–71. doi: 10.1021/acs.langmuir.1c03374 (PMC8867726; doi:10.1021/acs.langmuir.1c03374)
Supplement: Supplementary file 1 — la1c03374_si_001.pdf [file la1c03374_si_001.pdf]

SUPPORTING INFORMATION

Interferometry and simulation of the thin liquid film between a free-rising bubble and a glass substrate

Ivan U. Vakarelski,[1] Kenneth R. Langley[1,2], Fan Yang[1] and Sigurdur T. Thoroddsen[1]*

[1]Division of Physical Sciences and Engineering, King Abdullah University of Science and Technology (KAUST), Thuwal, 23955-6900, Saudi Arabia

[2]Department of Mechanical, Aerospace and Biomedical Engineering, University of Tennessee Space Institute, Tullahoma, TN, 37388, United States

* CORRESPONDING AUTHOR:

Ivan U. Vakarelski, E-mail: ivakarelski@gmail.com

Number of pages: #3

Number of figures: #2

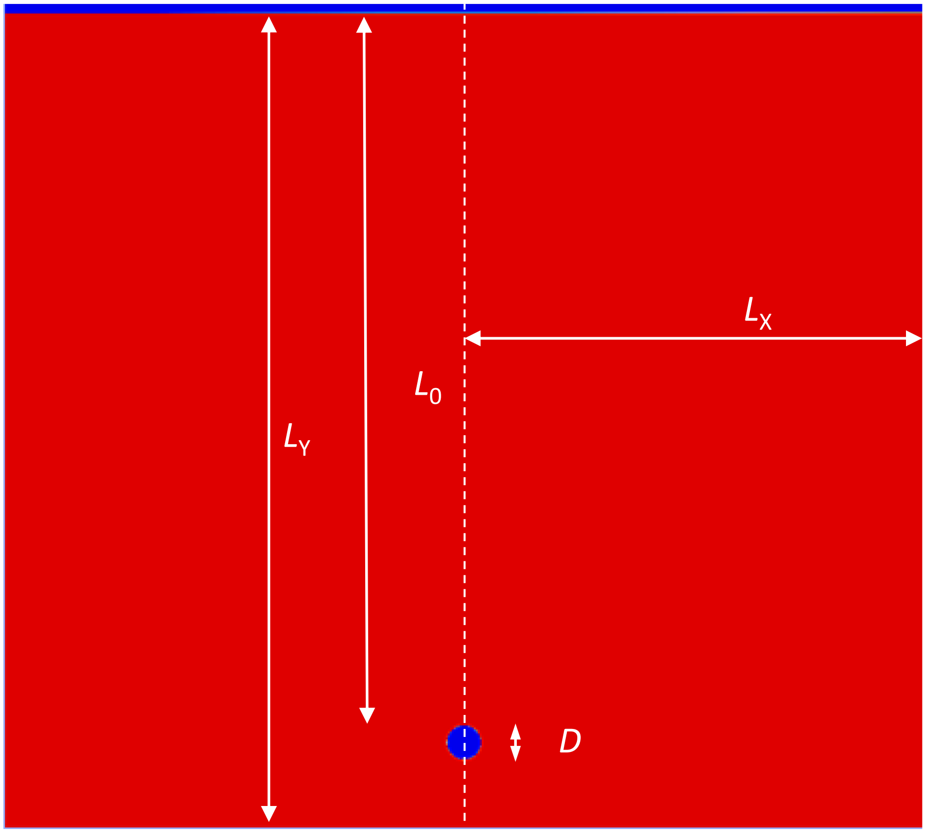

Supporting Figure S1. Computational domain with initial bubble position in the GNS of a bubble rising in water and rebounding from a top solid wall. Red is water liquid; blue circle is air bubble, blue line at the top is the wall. D = 1, L_x = 13.5, L_Y = 24, L_0 = 21. The domain of computation is limited to the left-hand side, due to the axisymmetric flow condition.

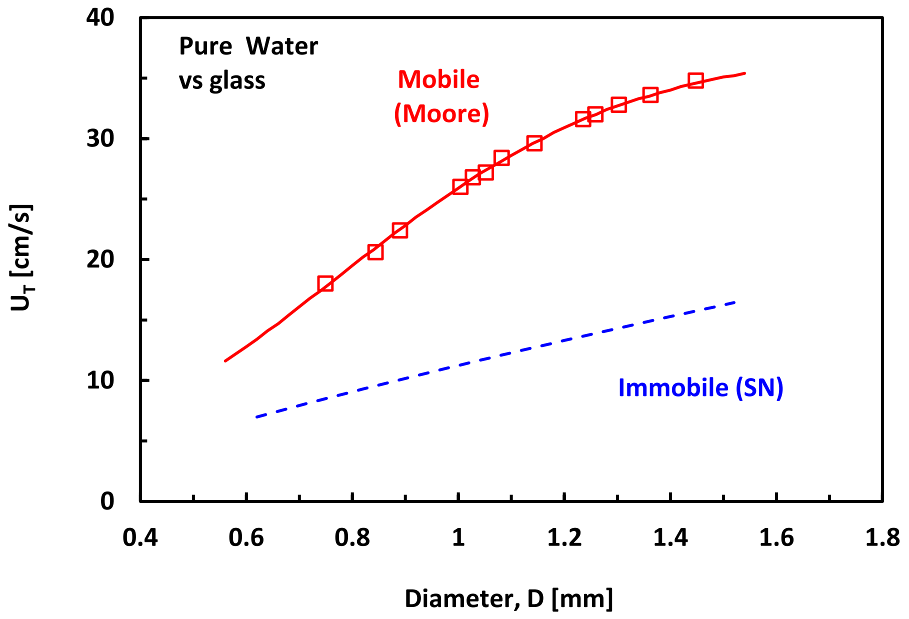

Supporting Figure S2. Measured terminal velocities, U_T of air bubbles free-rising in water versus a glass slide sample (empty squares, red) compared with the prediction of the Moore theory for mobile deformable bubbles in water (solid line, red).[S1,S2] The dashed lines are Schiller-Naumann dependence for immobile interface spherical bubbles in water (dashed line, blue). [S2]

REFERENCES

S1. Moore, D.W. The velocity of rise of distorted gas bubbles in a liquid of small viscosity. *J. Fluid Mech.* **1965**, *23*, 749-766.

S2. Manica, R.; Klaseboer, E.; Chan, D. Y. C. The impact and bounce of air bubbles at a flat fluid interface. *Soft Matter* **2016**, *12*, 3271-3282.
